# Prevention of haematoma progression by tranexamic acid in intracerebral haemorrhage patients with and without spot sign on admission scan: a statistical analysis plan of a pre-specified sub-study of the TICH-2 trial

**DOI:** 10.1186/s13104-018-3481-8

**Published:** 2018-06-13

**Authors:** Christian Ovesen, Janus Christian Jakobsen, Christian Gluud, Thorsten Steiner, Zhe Law, Katie Flaherty, Rob A. Dineen, Philip M. Bath, Nikola Sprigg, Hanne Christensen

**Affiliations:** 1 Department of Neurology, Bispebjerg Hospital, Copenhagen University Hospital, Bispebjerg bakke 23, 2400 Copenhagen, Denmark; 2 The Copenhagen Trial Unit, Centre for Clinical Intervention Research, Rigshospitalet, Copenhagen University Hospital, Copenhagen, Denmark; 3 Department of Cardiology, Holbæk Hospital, Holbæk, Denmark; 4 Department of Neurology, Klinikum Frankfurt Höchst, Frankfurt, Germany; 5 Department of Neurology, Heidelberg University Hospital, Heidelberg, Germany; 6 Stroke Trials Unit, Division of Clinical Neuroscience, University of Nottingham, City Hospital Campus, Nottingham, NG5 1PB UK; 7 Department of Medicine, National University of Malaysia, 56000 Kuala Lumpur, Malaysia; 8 Radiological Sciences, Division of Clinical Neuroscience, Queen’s Medical Centre, University of Nottingham, Nottingham, NG7 2UH UK; 9 Sir Peter Mansfield Imaging Centre, University of Nottingham, Nottingham, NG7 2QX UK; 10 NIHR Nottingham Biomedical Research Centre, Nottingham, NG1 5DU UK

**Keywords:** Intracerebral haemorrhage, Haematoma expansion, Tranexamic acid, Spot sign, Haemostatics

## Abstract

**Objective:**

We present the statistical analysis plan of a prespecified Tranexamic Acid for Hyperacute Primary Intracerebral Haemorrhage (TICH)-2 sub-study aiming to investigate, if tranexamic acid has a different effect in intracerebral haemorrhage patients with the spot sign on admission compared to spot sign negative patients. The TICH-2 trial recruited above 2000 participants with intracerebral haemorrhage arriving in hospital within 8 h after symptom onset. They were included irrespective of radiological signs of on-going haematoma expansion. Participants were randomised to tranexamic acid versus matching placebo. In this subgroup analysis, we will include all participants in TICH-2 with a computed tomography angiography on admission allowing adjudication of the participants’ spot sign status.

**Results:**

Primary outcome will be the ability of tranexamic acid to limit absolute haematoma volume on computed tomography at 24 h (± 12 h) after randomisation among spot sign positive and spot sign negative participants, respectively. Within all outcome measures, the effect of tranexamic acid in spot sign positive/negative participants will be compared using tests of interaction. This sub-study will investigate the important clinical hypothesis that spot sign positive patients might benefit more from administration of tranexamic acid compared to spot sign negative patients.

*Trial registration* ISRCTN93732214 (http://www.isrctn.com)

**Electronic supplementary material:**

The online version of this article (10.1186/s13104-018-3481-8) contains supplementary material, which is available to authorized users.

## Introduction

Limiting post-admission intraparenchymal haematoma expansion is one of the most promising targets for interventional research aiming to improve the functional outcome after intracerebral haemorrhage (ICH). Multiple studies have shown that intraparenchymal haematoma expansion contributes to the acute neurological instability of the patients [1–3] and poor long-term functional outcome [4, 5]. Data from the Intensive Blood Pressure Reduction in Acute Intracerebral Haemorrhage Trial (INTERACT) [5] estimated that post-admission intraparenchymal haematoma expansion increased the risk of death or dependency by 5% per millilitre additional haematoma volume. Hence limitation of intraparenchymal haematoma expansion would theoretically have a huge impact on outcome. In a population of patients with acute ICH, roughly 30% can be expected to undergo post-admission expansion of the intraparenchymal haematoma depending on the timing of the scans and the definition of expansion utilised [6].

**Fig. 1:**
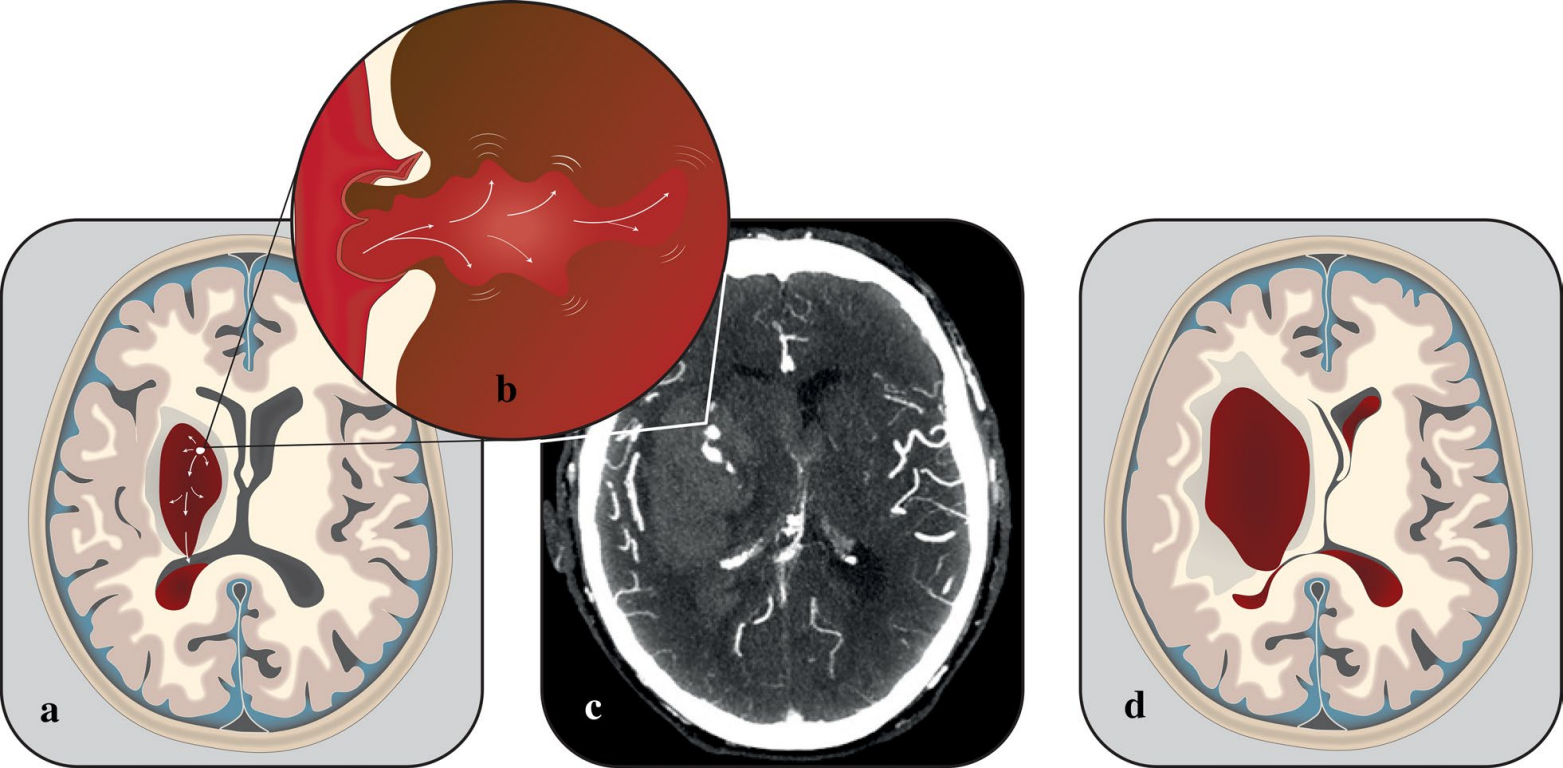
The spot sign is generally understood to be a radiological visualisation of an actively bleeding ruptured blood vessel (**b**) causing intraparenchymal haematoma expansion and by extension expansion of the intraventricular haematoma (**a**). The actively bleeding ruptured vessel is visualised on CT-angiography as the spot sign. **c** Depicts an authentic CT-angiography image of an acute intracerebral haemorrhage patient. A relatively large spot sign can be observed within the haematoma. Significantly enlarged intraparenchymal and intraventricular haematomas can often be seen on follow-up imaging (**d**)

Post-admission expansion of the intraparenchymal haematoma may not only cause additional damage to local brain parenchyma. On-going bleeding within the intraparenchymal haematoma may also cause expansion of pre-existing intraventricular haematoma by allowing additional blood to leak into the ventricular system [7] (Fig. 1). Alternatively, in patients with no intraventricular haematoma on admission, post-admission intraparenchymal haematoma expansion can result in decompression of the intraparenchymal haematoma into the ventricular system and the formation of a delayed intraventricular haematoma [8–10]. Consequently, impairing on-going bleeding within the intraparenchymal haematoma is likely to stabilise not only the intraparenchymal haematoma, but also the intraventricular haemorrhagic component. A similar mechanism can be assumed for subarachnoid haemorrhagic extension.

The proposed strategy used to stabilize the haematoma in several on-going trials is the acute administration of a haemostatic agent. The Tranexamic Acid For Hyperacute Primary Intracerebral Haemorrhage (TICH)-2 trial [11] aims to investigate, whether acute administration of tranexamic acid can improve functional outcome after ICH. Previous trials on haemostatic agents in patients with acute ICH have shown that haematoma expansion can be significantly inhibited; however, no improvement in functional outcome has been shown [12]. It has been hypothesized that selecting patients with radiological signs of on-going haematoma expansion is essential, as only patients with on-going haematoma expansion can be expected to benefit from the administration of haemostatic agents. On the other hand, patients without on-going haematoma expansion may not benefit from a more stable haematoma, potentially leaving this patient-group with only the risk of adverse events [13].

Even though the safety profile of tranexamic acid has in general been found to be good, one of the greatest theoretical concerns when administering a haemostatic agent is the risk of a thromboembolic event. The large CRASH-2 trial [14] did not confirm an increased risk of thromboembolic events in the group treated with tranexamic acid among acute trauma patients. However, previous trials randomising intracerebral haemorrhage patients to recombinant factor VII did observe an increased risk of thromboembolic events related to the treatment [12]. In addition, observational studies of intracerebral haemorrhage patients have observed a relative high prevalence of cerebral ischemic lesions, when the patients are scanned in the sub-acute period after symptom onset [15, 16]. This finding could indicate that the risk of cerebral ischemia could be relatively high in acute intracerebral haemorrhage patients suggesting a potential added risk of administering haemostatic agents.

The TICH-2 trial includes acute ICH patients regardless of radiological signs of on-going bleeding. The present manuscript details a pre-specified sub-study of the TICH-2 trial aiming to explore, whether contrast enhanced radiological signs of on-going haematoma expansion can identify patients, who will benefit more from the administration of tranexamic acid, and whether patients with radiologically stable haematomas risk harm by adverse effects without the compensating benefit of limited haematoma expansion. In order to identify participants with on-going haematoma expansion on admission, we will utilise the spot sign based on computed tomography angiography (CTA).

The predictive capability of the spot sign towards haematoma expansion has been confirmed in many studies. It has been hypothesized that the spot sign represents active leakage of blood from vessels adjacent to the haematoma and consequently represents on-going haematoma expansion (Fig. 1). In general, the specificity of the spot sign is reported as good (meta-analysed specificity 0.88, confidence interval (CI) 0.86–0.89), whereas the sensitivity is reported as fair (meta-analysed sensitivity 0.53, CI 0.49–0.57) [17]. Two separate systematic reviews have concluded that inferences regarding the reliability of the spot sign to predict haematoma expansion could be influenced by poor or heterogeneous methodology and possibly publication bias [17, 18]. The scan protocol used when conducting CTA can also significantly influence the prevalence of the spot sign and its predictive capability towards haematoma expansion [19]. Spot signs observed in the venous phase are more prevalent, but its prediction towards haematoma expansion is poorer compared to spot signs observed in the arterial phase. Besides the spot sign on CTA, extravasation of contrast on post-contrast computed tomography can predict haematoma expansion even in spot sign negative patients [20, 21]. Even though the spot sign, like all other biomarkers, has its limitations, it remains the most widely investigated and clinically established predictor of on-going haematoma expansion in acute intracerebral haemorrhage patients.

### Primary research question

This TICH-2 sub-study aims to investigate, whether the spot sign on CTA can identify patients with a high chance of benefit from acute administration of tranexamic acid.

### Main trial design

The present manuscript describes a pre-specified sub-group study of the TICH-2 trial (http://www.tich-2.org).

The TICH-2 trial is an international placebo-controlled, blinded, randomised trial aiming at determining, whether administration of tranexamic acid versus placebo to participants with spontaneous intracerebral haemorrhage is able to reduce death and dependency at 3 months.

The protocol for the TICH-2 trial has already been published [11]. In brief, the TICH-2 trial randomises adult patients with acute spontaneous ICH arriving in hospital within 8 h after stroke onset to either 2 g tranexamic acid versus matching placebo. The participants are randomised after diagnostic non-contrast computed tomography (CT) scan (and optionally CTA) has revealed a spontaneous intracerebral haemorrhage. Participants are randomised in a 1:1 ratio using an adaptive randomisation procedure minimising differences between treatment arms with regard to age, sex, time from onset to randomisation, mean systolic blood pressure, stroke severity, presence of intraventricular haemorrhage, and known history of antiplatelet treatment. The randomisation procedure stratifies the allocation for country.

Two grams of tranexamic acid or matching placebo is administered as an intravenous infusion over 8 h. The participant is then observed and treated in the hospital as per local guidelines. Twenty-four hours (± 12 h) after admission, a 24-h CT will provide the final haematoma volume. After a period of 3 months, the participant/caregiver will be contacted by telephone in order to assess the participant’s functional outcome/mortality.

The TICH-2 trial will include at least 2000 participants. The primary outcome of the TICH-2 trial is death or dependency at day 90.

## Main text

### Analysis population and missing data

The TICH-2 main population will be analysed using the intention-to-treat principle. All randomised participants will be included in the present sub-group analysis, provided they have undergone an admission CTA allowing the spot sign status to be assessed and not having withdrawn consent within the first 24 h. The sample size of this sub-study will be determined by enrolment in the TICH-2 study. Missing minimisation criteria and missing data will be handled as indicated in the TICH-2 main statistical analysis plan (SAP) [22].

### Outcomes

For all outcome analysis, the population of participants will be separated into two subgroups—with and without spot sign on CTA. Within each of the spot sign groups, we will compare the mean difference/probability of the outcome between participants allocated to tranexamic acid versus placebo adjusting for minimisation and stratification factors. The homogeneity of the effect estimates (spot sign positive compared to spot sign negative) will be analysed by interaction-tests.

#### Primary outcome

Absolute intraparenchymal haematoma volume on 24-h (± 12 h) CT (or if 24-h CT is not available or biased, a CT obtained after randomisation but before the day-2 scan window will be used). The primary outcome will also be analysed as intraparenchymal haematoma combined with intraventricular haematoma.

All randomised participants will be included in the primary analysis, provided that they have a 24-h CT performed (or if 24-h CT is not available or biased, a CT obtained after randomisation but before the 24-h (± 12 h) scan window will be used). Biased 24-h CT will be defined as a 24-h CT obtained after surgical procedures aiming at total or partial removal of intraparenchymal or intraventricular haematoma.

#### Secondary outcomes

• Dichotomous haematoma progression defined as a composite outcome. The proposed dichotomous composite outcome is necessary in order to avoid bias by limiting the population to participants fit enough to have a per-protocol 24-h CT performed. It is likely that a considerable group will either die or undergo surgical procedures, prior to the per-protocol 24-h CT can be obtained. It is further likely that this group will not be distributed at random yielding a large risk of bias and reduction of external validity.
  The following will constitute haematoma progression on 24-h CT (± 12 h) relative to the admission CT:
  ∘ Intraparenchymal haematoma expansion (≥ 6 mL absolute expansion or/and 33% relative expansion), and/or
  ∘ Delayed intraventricular or subarachnoid haemorrhagic extension, and/or
  ∘ Intraventricular haematoma expansion (≥ 2 mL absolute expansion).
  In case the per-protocol 24-h CT is biased (defined as partial or complete surgical removal of haematoma) or not available (caused by do-not-resuscitate orders, withdrawal-of-care orders, death, or protocol violation), the following will be classified as haematoma progression in the outcome analysis:
  ∘ Expansion of either intraventricular or intraparenchymal haematoma (as defined above) or delayed intraventricular or subarachnoid haemorrhagic on emergency CT obtained after randomisation but before the 24-h (± 12 h) scan time window (the same definitions of expansion will apply, as if they were observed on the per-protocol 24-h CT), and/or
  ∘ Early neurological deterioration or death between admission and the day-2 clinical assessment.
• In order to ascertain, if the treatment effect measured by the composite outcome is being driven be any of the different components in particular, analysis of the individual components will be conducted.
• Serious adverse event within the first 7 days after randomisation.
• Safety outcome within the first 90 days after randomisation.
• Thromboembolic event within the first 90 days after randomisation.
• Day-90 modified Rankin scale will be analysed as a dichotomous poor outcome (modified Rankin Scale 4–6).
• Day-90 Barthel Index will be analysed as a continuous outcome.
• Mortality at day-90.

Please confer definitions listed in Additional file 1.

### Power estimations

As acute CTA does not represent standard-of-care in the evaluation of ICH-patients in all countries, only a subpopulation of randomised participants in the TICH-2 trial can be expected to have had one performed. We estimate that approximately 60 spot sign positive participants (30 allocated to tranexamic acid and 30 to placebo) will be included. We also estimate that 10% will have missing or biased 24-h CT. This yields a probability of rejecting the primary outcome null hypothesis [mean difference (MD) 14 mL, standard deviation (SD) 17 mL, α-level 0.05] [23] that tranexamic acid does not prevent haematoma expansion in spot sign positive participants of 84%.

We also estimate that approximately 180 spot sign negative participants will be included (90 allocated to tranexamic acid and 90 to placebo). We estimate that 10% will have missing 24-h CT. This yields a probability of rejecting the primary outcome null hypothesis (MD 2 mL, SD 8 mL, α-level 0.05) [23] that tranexamic acid does not prevent haematoma expansion in spot sign negative participants of 35%.

In order to estimate the power of the test of interaction between spot sign status and allocation (tranexamic acid versus placebo), we used the user written program ‘Powersim’ [24]. We used a MD of 12 mL and a SD of 14 mL for all effects, together with a risk of type 1 error of 5%, which resulted in an estimated power of 85%. Power calculations for secondary outcomes are presented in Additional file 2.

### Statistical methodology

Regression models will be used to analyse primary and secondary outcomes in order to allow for adjustment whenever appropriate. For all outcomes, an effect size (tranexamic acid versus placebo) will be calculated for participants with and without spot sign on admission. The two effect sizes will be compared using a test of interaction. Multivariable analyses will be undertaken adjusting effect estimates for minimization and stratification factors. As the sample of especially spot sign positive participants is relatively small, we recognise the potential risk of over-fitting the model, if the models are adjusted for all minimization factors. If such a situation arrives, we will limit the number of covariates in the analysis to those of particular importance including age, time from stroke onset to treatment, and stroke severity (admission National Institute of Health Stroke Scale), as these have been consistently shown to be related to outcome [13, 25]. The number of observations deemed necessary in order to reduce the risk of over-fitting the models is presented in Additional file 3. We will consequently present a set of analyses adjusted for the at least three above-mentioned minimization factors, and if sample size permits, another set of analysis will be provided with all the minimisation factors included. The model building will be discussed in final publication.

The following models will be used:

• In analysing the primary outcome, the main parameter of interest will be the mean difference in absolute haematoma volume on 24-h (± 12 h) CT between participants randomised to tranexamic acid versus placebo adjusted for baseline haematoma volume [26, 27]. This outcome will be analysed using multiple linear regression.
• The secondary composite outcome, serious adverse events, safety events and thromboembolic event will be analysed using binary logistic regression.
• Day-90 Barthel Index will be analysed using multiple linear regression.
• Day-90 dichotomous modified Rankin scale will be analysed using binary logistic regression.
• Mortality within the first 90-days will be analysed using Cox proportional hazard model.

An important assumption in statistical procedures is independence of observations. This assumption is often violated, as correlation often exists between clusters of participants, in which participants share more characteristics than participants between clusters [28]. This is especially the case, when randomisation procedures stratify the allocation according to some entity—e.g. hospital or country. Overlooking this correlation structure can lead to too narrow confidence intervals [28, 29]. In the supplements of our final report, we will repeat the above specified analysis using generalized estimating equation (linear and logistic) with non-robust standard error [29, 30] in order to account for the cluster-effect of country as the minimization procedure was stratified for country. Differences in reached conclusions will be discussed.

For all regression models used, we will carefully report, how fulfilment of underlying assumptions was assessed. The statistical assumptions are presented in Additional file 3.

### Statistical inferences

Oxman et al. [31] proposed a number of stipulations guiding the trustworthiness of sub-group analysis of randomised trials. This TICH-2 subgroup analysis is a pre-specified analysis, which underlying hypothesis is supported by our pathophysiological understanding of the spot signs predictive capability towards haematoma expansion. An interaction between the spot sign status and the pharmacological effect of tranexamic acid in limiting the primary outcome is an important clinical hypothesis and can ideally be studied in this setting, as both spot sign positive and negative participants are treated within the same trial and hence following the same protocol [31]. This increases the validity of the clinical and statistical inferences made on the basis of this analysis.

In this study, an analysis will be considered statistically significant, if the probability of type 1 error is less than 5% (2-sided). As we recognise that the statistical power of the interaction-test testing the homogeneity of the treatment response between participants with and without spot sign will likely have a low power, the inferences drawn from this sub-group analysis will be exploratory in nature [32, 33]. Simulation-studies have shown that inflation of the original sample size is often necessary to maintain the power of the main primary outcome (often 80 or 90%) in the sub-group analysis [33]—hence this sub-group analysis will theoretically contain an enlarged risk of false-negative findings, even though simulation-based power analysis indicates an acceptable power for the primary outcome of this sub-group analysis. To obtain a sufficient power, the present sub-group analysis might need to be included in meta-analysis with other on-going studies. At least two other trials (STOP-AUST and TRAIGE) are currently randomising spot sign positive participants with intracerebral haemorrhage to either tranexamic acid or placebo (ClinicalTrial.gov NCT01702636 and NCT02625948, accessed November 2017) [34–36].

For the secondary outcomes, the risk of multiplicity implies. In case of an insignificant primary outcome in both participants with and without spot sign on admission scan, no effort will be undertaken to control the inflation of type 1 error due to multiplicity in the secondary outcomes, as no isolated direct clinical inferences will be drawn from them—however, their significance will be discussed.

After the statistical analysis, we will thoroughly assess the clinical significance of the results [37].

## Definitions

Definitions are presented in Additional file 1.

## Pre-planned tables and figures

Pre-planned tables and figures are presented in Additional file 4.

## Discussion

We have above presented the statistical analysis plan for the spot sign sub-study of the TICH-2 trial. As elaborated, such sub-studies might have archived a bad reputation for being potentially misleading, data-driven, and underpowered [31]. However, we believe that since the underlying hypothesis is supported by our pathophysiological understanding of the spot sign, the sub-group analysis is pre-planned, and the methodology is sought to be as rigorous as possible, we will be able to archive methodological quality in our analysis. Currently, other trials are randomising spot sign positive ICH patients to tranexamic acid versus placebo [34–36]. The data of this subgroup-analysis will be highly interesting for inclusion in meta-analysis with these forthcoming trials.

The primary outcome will be mean difference in 24-h absolute haematoma volume compared between participants allocated to tranexamic acid versus placebo, adjusted for haematoma volume at baseline. We choose to analyse the 24-h absolute haematoma volume (adjusted for baseline haematoma volume) instead of analysing a measure of change from baseline (e.g. millilitre haematoma expansion or per cent volume expansion). Studies have shown that measures of change can potentially be biased, if baseline differences in haematoma volume exist between treatment groups and that measures of change can yield lower statistical power [26, 27]. The continuous outcome of 24-h absolute haematoma volume was chosen as the primary outcome as this outcome yielded the highest calculated statistical power. However, due to the poor clinical condition of a relatively large proportion of intracerebral haemorrhage patients, it must be expected that some of the participants in the trial will have missing day-2 scans excluding them from the primary outcome analysis. Anticipating this, we designed the secondary composite outcome of haematoma progression.

## Limitations

As discussed above, one should always be cautious in drawing firm conclusions from sub-studies, especially when it comes to making clinical recommendations. A clear limitation of this study is its possibility of generating results of low external validity. Participants are recruited into this sub-study, based on whether they have had a CT-angiography performed on admission. As the CT-angiography is not standard-of-care in many centres, it is likely that the participants, who had a CT-angiography performed, are systematically different from the rest of the TICH-2 population. We believe that this fact emphasises that inferences drawn from this study need confirmation in other trials. It is consequently the hope of the authors that this sub-study can be hypothesis-generating, and can inform future trialists when designing trials assessing the effect of tranexamic acid in intracerebral haemorrhage patients.

## Additional files

**Additional file 1.** Study definitions—definitions used in the planed analysis.

**Additional file 2.** Power calculations—power calculations of primary and secondary outcomes.

**Additional file 3.** Statistical assumptions—statistical assumptions of the regression models.

**Additional file 4.** Preplanned tables and figures.

## Abbreviations

TICH: Tranexamic Acid for Hyperacute Primary Intracerebral Haemorrhage
ICH: intracerebral haemorrhage
CT: computed tomography
CTA: computed tomography angiography
SAP: statistical analysis plan
