# Supplementary material for: Prevention of haematoma progression by tranexamic acid in intracerebral haemorrhage patients with and without spot sign on admission scan: a statistical analysis plan of a pre-specified sub-study of the TICH-2 trial
Source: BMC Res Notes. 2018 Jun 13;11:379. doi: 10.1186/s13104-018-3481-8 (PMC5998558; doi:10.1186/s13104-018-3481-8)
Supplement: Supplementary file 1 — Additional file 1. Study definitions—definitions used in the planed analysis. [file 13104_2018_3481_MOESM1_ESM.docx]

**STUDY DEFINITIONS**

Definitions below is harmonised with the TICH-2 main statistical analysis plan (SAP) [1].

Definitions of the spot sign: In primary data analysis, participants CTA will be evaluated for spot sign status by a blinded radiological observer using the following definition. The definition of the spot sign is adopted from the PREDICT/Sunnybrook ICH CTA study group [2]:

- **Appearance:** Serpiginous and/or spot-like appearance.
- **Location:** Within the margin of the parenchymal haematoma without connection to an outside vessel.
- **Size:** > 1.5 mm in diameter in the maximal axial dimension.
- **Density:** At least double to density (Hounsfield unit) compared to background haematoma.
- **Lesion number:** Multiple or single**.**

The definition described above is adopted, as it has been shown to provide good predictive capabilities towards haematoma expansion [3]. A participant will be classified as spot sign positive, if at least one spot sign satisfies the above-mentioned criteria regardless of the contrast phase on CTA, in which it is observed, or any other local CTA settings or protocols utilized. Before a patient is allocated as spot sign positive, the pre-contrast CT should be reviewed to avoid misinterpretation of calcification-mimics [4].

If post-contrast imaging is obtained, patients will also be classified as spot sign positive if: at least one hyperdensity (relative to the haematoma) within the haematoma can be demonstrated indicative of contrast extravasation on post-contrast imaging (not present on pre-contrast CT) [5].

Due to the expected heterogeneous methodology concerning CT-angiography and post-contrast imaging among centres, we plan to conduct the following sensitivity analyses. In sensitivity data-analyses, participants will be analysed according to the spot sign status (spot sign positive or negative):

- On CTA only (excluding post-contrast sequences).
- As adjudicated by the local investigator during the randomisation procedure based on emergency CTA (± post contrast sequences).

Intraparenchymal haematoma expansion: Volume of the intraparenchymal haematoma will be calculated separately on the admission and the 24 hour CT (± 12 hours) (or CT obtained earlier, if per-protocol 24-hour CT is missing or biased) using computer assisted semi-automated segmentation. The absolute haematoma expansion is calculated as the difference between the two CTs. Intraparenchymal haematoma expansion will be defined as relative growth of at least 33% or an absolute growth of ≥6 mL.

Intraventricular haematoma expansion: Volume of the intraventricular haematoma will be calculated separately on the admission and the 24-hour CT (± 12 hours) (or CT obtained earlier, if per-protocol 24-hour CT is missing or biased) using computer assisted semi-automated segmentation. Delayed intraventricular haemorrhage is defined as intraventricular haemorrhage not present on admission imaging but appearing on follow-up imaging. The absolute intraventricular haematoma expansion is calculated as the difference between the admission and day-2 intraventricular haemorrhagic volumes (if no intraventricular haematoma is present on admission, the volume will be adjudicated as zero). Intraventricular haematoma expansion is defined as an absolute volume increase of 2 mL [6].

Delayed subarachnoid haemorrhagic extension: Decompression of the intraparenchymal haematoma into the subarachnoid space on 24-hour CT (or CT obtained earlier, if per-protocol 24-hour CT is missing or biased) but not on admission CT.

Serious adverse event: Adverse events will be defined using the general Good Clinical Practice-criteria (ICH-GCP E6, www.ich.org) and the criteria outlined in the TICH-2 trial protocol [1].

Safety outcome: Defined as thromboembolism (please confer definition below), seizures, or death within 90 days. Definitions of seizures are presented in the TICH-2 main SAP [1].

Thromboembolism: Thromboembolism will be defined as either unstable angina, ST-segment elevation myocardial infarction (STEMI) or non-ST-segment elevation myocardial infarction (NSTEMI), ischaemic stroke, transient ischaemic attack, peripheral arterial disease, deep vein thrombosis, or pulmonary embolism within the first 90 days. The definitions of each of these events are presented in the TICH-2 main SAP [1].

Early neurological deterioration: Early neurological deterioration (deterioration between admission and day-2 assessment) is defined as either:

- A day-2 worsening in neurological performance (increase of at least 4 National Institute of Health Stroke Scale (NIHSS) points) or consciousness (decrease of 2 Glasgow Coma Scale (GCS) points); levels obtained at randomisation is reference.
- A decrease in neurological performance or consciousness (within the first 24 hours) leading to intubation or neurosurgical intervention documented in a serious adverse event report.
- Death during the first 24 hours.

**SUPPLEMENTARY REFERENCES**

1 Flaherty K, Bath PM, Dineen R, Law Z, Scutt P, Pocock S, Sprigg N, investigators T-: Statistical analysis plan for the 'tranexamic acid for hyperacute primary intracerebral haemorrhage' (tich-2) trial. Trials 2017;18:607.

2 Thompson AL, Kosior JC, Gladstone DJ, Hopyan JJ, Symons SP, Romero F, Dzialowski I, Roy J, Demchuk AM, Aviv RI, Group PRSICS: Defining the ct angiography 'spot sign' in primary intracerebral hemorrhage. Can J Neurol Sci 2009;36:456-461.

3 Demchuk AM, Dowlatshahi D, Rodriguez-Luna D, Molina CA, Blas YS, Dzialowski I, Kobayashi A, Boulanger JM, Lum C, Gubitz G, Padma V, Roy J, Kase CS, Kosior J, Bhatia R, Tymchuk S, Subramaniam S, Gladstone DJ, Hill MD, Aviv RI, group PRSICs: Prediction of haematoma growth and outcome in patients with intracerebral haemorrhage using the ct-angiography spot sign (predict): A prospective observational study. Lancet Neurol 2012;11:307-314.

4 Gazzola S, Aviv RI, Gladstone DJ, Mallia G, Li V, Fox AJ, Symons SP: Vascular and nonvascular mimics of the ct angiography "spot sign" in patients with secondary intracerebral hemorrhage. Stroke 2008;39:1177-1183.

5 Hallevi H, Abraham AT, Barreto AD, Grotta JC, Savitz SI: The spot sign in intracerebral hemorrhage: The importance of looking for contrast extravasation. Cerebrovasc Dis 2010;29:217-220.

6 Steiner T, Diringer MN, Schneider D, Mayer SA, Begtrup K, Broderick J, Skolnick BE, Davis SM: Dynamics of intraventricular hemorrhage in patients with spontaneous intracerebral hemorrhage: Risk factors, clinical impact, and effect of hemostatic therapy with recombinant activated factor vii. Neurosurgery 2006;59:767-773.
