# Supplementary material for: Prevention of haematoma progression by tranexamic acid in intracerebral haemorrhage patients with and without spot sign on admission scan: a statistical analysis plan of a pre-specified sub-study of the TICH-2 trial
Source: BMC Res Notes. 2018 Jun 13;11:379. doi: 10.1186/s13104-018-3481-8 (PMC5998558; doi:10.1186/s13104-018-3481-8)
Supplement: Supplementary file 2 — Additional file 2. Power calculations—power calculations of primary and secondary outcomes. [file 13104_2018_3481_MOESM2_ESM.docx]

**POWER CALCULATIONS**

Below are listed power calculations for all primary and secondary outcomes

|  | **Spot sign positive** | **Spot sign negative** |
| --- | --- | --- |
| Primary continuous outcome | Approximate number of patients available: 54 (27 in each allocation group). We have assumed that 10% will have missing day-2 CTC.    We assume that the absolute haematoma expansion volume within each group is normally distributed with a standard deviation of 17 mL. We also assume that the true mean difference is 14 mL.[1]  **Probability of rejecting the null hypothesis (power) .844.** | Approximate number of patients available: 162 (81 in each allocation group). We have assumed that 10% will have missing day-2 CTC.    We assume that the absolute haematoma expansion volume within each group is normally distributed with a standard deviation of 8 mL. We also assume that the true mean difference is 2 mL.[1]  **Probability of rejecting the null hypothesis (power) .351.** |
| Secondary composite binary outcome | Approximate number of patients available: 60 (30 in each allocation group).    We assume an event-rate in the control-population of 50%.[2] We also assume a RRR of 40%.  **Probability of rejecting the null hypothesis (power) .349**. | Approximate number of patients available: 180 (90 in each allocation group).    We assume an event-rate in the control-population of 10%.[2] We also assume a RRR of 40%.  **Probability of rejecting the null hypothesis (power) .167** |
| Serious adverse events within the first 7 days | Approximate number of patients available: 60 (30 in each allocation group).  We assume an event-rate in the control population of 50%. We also assume a RRI of 20%.  **Probability of rejecting the null hypothesis (power) .120.** | Approximate number of patients available: 180 (90 in each allocation group).  We assume an event-rate in the control population of 50%. We also assume a RRI of 20%.  **Probability of rejecting the null hypothesis (power) .270.** |
| Safety outcome within the first 90 days | Approximate number of patients available: 60 (30 in each allocation group).  We assume an event-rate in the control population of 50%. We also assume a RRI of 20%.  **Probability of rejecting the null hypothesis (power) .120.** | Approximate number of patients available: 180 (90 in each allocation group).  We assume an event-rate in the control population of 30%. We also assume a RRI of 20%.  **Probability of rejecting the null hypothesis (power) .137.** |
| Thromboembolic events within the first 90 days | Approximate number of patients available: 60 (30 in each allocation group).  We assume an event-rate in the control population of 10%.[3] We also assume a RRI of 20%.  **Probability of rejecting the null hypothesis (power) .057.** | Approximate number of patients available: 180 (90 in each allocation group).  We assume an event-rate in the control population of 10%.[3] We also assume a RRI of 20%.  **Probability of rejecting the null hypothesis (power) .071.** |
| 90-day functional outcome (modified Rankin Scale) - dichotomised | Approximate number of patients available: 60 (30 in each allocation group).    We assume an event-rate in the control-population of 70%. [4,5] We also assume a RRR of 20%.  **Probability of rejecting the null hypothesis (power) .200** | Approximate number of patients available: 180 (90 in each allocation group).    We assume an event-rate in the control-population of 50%. [4,5] We also assume a RRR of 20%.  **Probability of rejecting the null hypothesis (power) .270.** |
| 90-day functional outcome (Barthel index) - continuous | As no previous study using the CTA-based spot sign has published data on Barthel-index, reliable power-calculation could not be conducted. | As no previous study using the CTA-based spot sign has published data on Barthel-index, reliable power-calculation could not be conducted. |
| 90-day survival outcome | Approximate number of patients available: 60 (30 in each allocation group). We estimate an accrual interval of 1 time units and additional follow-up after the accrual interval of 89 time units.    In a previous study, the median survival time on the control treatment was approximately (extrapolated[6]) 174.9 time units.[2]  We assume a true hazard ratio of control subjects relative to experimental subjects is 0.8,  **Probability of rejecting the null hypothesis (power) .078.** | Approximate number of patients available: 180 (90 in each allocation group). We estimate an accrual interval of 1 time units and additional follow-up after the accrual interval of 89 time units.    In a previous study, the median survival time on the control treatment was approximately (extrapolated[6]) 384.0 time units.[2]  We assume a true hazard ratio of control subjects relative to experimental subjects is 0.8,  **Probability of rejecting the null hypothesis (power) .093.** |

For all power-calculations above, a probability of type 1 error of 5% is assumed. For dichotomous outcomes, power is calculated based on uncorrected chi^2^-test. Power-calculation was conducted using PS: Power and Sample size calculation version 3.1.2, 2014.[7] mL – millilitre; RRR – relative risk reduction; RRI – relative risk increase.

**SUPPLEMENTARY REFERENCES:**

1 Wada R, Aviv RI, Fox AJ, Sahlas DJ, Gladstone DJ, Tomlinson G, Symons SP: Ct angiography "spot sign" predicts hematoma expansion in acute intracerebral hemorrhage. Stroke 2007;38:1257-1262.

2 Demchuk AM, Dowlatshahi D, Rodriguez-Luna D, Molina CA, Blas YS, Dzialowski I, Kobayashi A, Boulanger JM, Lum C, Gubitz G, Padma V, Roy J, Kase CS, Kosior J, Bhatia R, Tymchuk S, Subramaniam S, Gladstone DJ, Hill MD, Aviv RI, group PRSICs: Prediction of haematoma growth and outcome in patients with intracerebral haemorrhage using the ct-angiography spot sign (predict): A prospective observational study. Lancet Neurol 2012;11:307-314.

3 Mayer SA, Brun NC, Begtrup K, Broderick J, Davis S, Diringer MN, Skolnick BE, Steiner T, Investigators FT: Efficacy and safety of recombinant activated factor vii for acute intracerebral hemorrhage. N Engl J Med 2008;358:2127-2137.

4 Delgado Almandoz JE, Yoo AJ, Stone MJ, Schaefer PW, Oleinik A, Brouwers HB, Goldstein JN, Rosand J, Lev MH, Gonzalez RG, Romero JM: The spot sign score in primary intracerebral hemorrhage identifies patients at highest risk of in-hospital mortality and poor outcome among survivors. Stroke 2010;41:54-60.

5 Rizos T, Dorner N, Jenetzky E, Sykora M, Mundiyanapurath S, Horstmann S, Veltkamp R, Rohde S, Bendszus M, Steiner T: Spot signs in intracerebral hemorrhage: Useful for identifying patients at risk for hematoma enlargement? Cerebrovasc Dis 2013;35:582-589.

6 Schoenfeld DA, Richter JR: Nomograms for calculating the number of patients needed for a clinical trial with survival as an endpoint. Biometrics 1982;38:163-170.

7 Dupont WD, Plummer WD, Jr.: Power and sample size calculations. A review and computer program. Control Clin Trials 1990;11:116-128.
