# Supplementary material for: Prevention of haematoma progression by tranexamic acid in intracerebral haemorrhage patients with and without spot sign on admission scan: a statistical analysis plan of a pre-specified sub-study of the TICH-2 trial
Source: BMC Res Notes. 2018 Jun 13;11:379. doi: 10.1186/s13104-018-3481-8 (PMC5998558; doi:10.1186/s13104-018-3481-8)
Supplement: Supplementary file 3 — Additional file 3. Statistical assumptions—statistical assumptions of the regression models. [file 13104_2018_3481_MOESM3_ESM.docx]

**STATISTICAL ASSUMPTIONS**

|  | **Statistical assumptions** | **How the assumption is reviewed** |
| --- | --- | --- |
| Multiple linear regression | 1: Independence of observations  2: Linearity of continuous covariates  3: Homoscedasticity  4: Normality  5: Sample size | 1: Assumed not violated for primary analysis.[1]  A sensitivity analysis will be presented, in which we will attempt to account for the correlation of participants recruited within the same country (stratification variable).  2: Scatterplots of jack-knife residuals plotted against continuous covariates as well as the predicted values. Each should follow a horizontal line. [1] This assumption can also be assessed using fractional polynomial analysis. [2]  3: Scatterplots of jack-knife residuals plotted against predicted values. Residual variance should be approximately constant across predicted values. [1]  4: Jack-knife residuals assessed by Q-Q plot. Points should follow diagonal line. [1]  5: Approximately 10-15 observations per covariate will be regarded as sufficient seen as ideal in order to avoid over-fitting the model. [1] |
| Logistic regression | 1: Independence of observations  2: Linearity of logit for continuous covariates  3: Sample size | 1: Assumed not violated for primary analysis. A sensitivity analysis will be presented, in which we will attempt to account for the correlation of participants recruited within the same country (stratification variable).  2: Will be assessed using locally weighted scatterplot smoothing (should follow a relatively straight line) and/or by using fractional polynomial analysis. [2,3]  3: Approximately 10 events per independent covariate will be seen as the ideal. However, even a lower number of outcome events per predictor variable (EPV) has been shown to be acceptable in terms of inflation of type 1 error and bias. However, caution in interpretation might be warranted with a low number of EVP. [4] |
| Cox proportional hazards model | 1: Independence of observations  2: Independent censoring  3: Linearity of log-hazard for continuous covariates  4: Proportional hazard  5: Sample size | 1: Assumed not violated for primary analysis.  2: This assumption will be assumed fulfilled. [5,6]  3: Will be assessed using Martingale residuals plotted against continuous covariate. [7] Fractional polynomial analysis can also be used. [2]  4: Will be assessed using scaled Schoenfeld residuals. If the calculated smoothed average is horizontal, proportional hazard is assumed for the covariate in question. A global test can be applied. [8,9]  5: Approximately 10 events per independent covariate will be seen as the ideal. However, even a lower number of outcome events per predictor variable (EPV) has been shown to be acceptable in terms of inflation of type 1 error and bias. However, caution in interpretation might be warranted with a low number of EVP. [4] |

**SUPPLEMENTARY REFERENCES:**

1 Kleinbaum DG, Kleinbaum DG: Applied regression analysis and other multivariable methods, ed 4th. Belmont, CA, Brooks/Cole, 2008.

2 Royston P, Altman DG: Regression using fractional polynomials of continuous covariates: Parsimonious parametric modelling. Journal of the Royal Statistical Society 1994;43:429-467.

3 Hosmer DW, Lemeshow S, Sturdivant RX: Applied logistic regression, ed 3rd. Hoboken, New Jersey, USA, John Wiley and Sons, 2013.

4 Vittinghoff E, McCulloch CE: Relaxing the rule of ten events per variable in logistic and cox regression. Am J Epidemiol 2007;165:710-718.

5 Austin PC, Lee DS, Fine JP: Introduction to the analysis of survival data in the presence of competing risks. Circulation 2016;133:601-609.

6 Jackson D, White IR, Seaman S, Evans H, Baisley K, Carpenter J: Relaxing the independent censoring assumption in the cox proportional hazards model using multiple imputation. Stat Med 2014;33:4681-4694.

7 Therneau TM, Grambsch PM, Fleming TR: Martingale-based residuals for survival models. Biometrika 1990;77:147-160.

8 Juul S, Frydenberg M: An introduction to stata for health researchers, ed 4th. College Station, Texas, StataCorp LP, 2014.

9 Machin D, Cheung YB, Parmar MKB: Survival analysis : A practical approach, ed 2nd. Chichester, Wiley, 2006.
