# Supplementary material for: Prevention of haematoma progression by tranexamic acid in intracerebral haemorrhage patients with and without spot sign on admission scan: a statistical analysis plan of a pre-specified sub-study of the TICH-2 trial
Source: BMC Res Notes. 2018 Jun 13;11:379. doi: 10.1186/s13104-018-3481-8 (PMC5998558; doi:10.1186/s13104-018-3481-8)
Supplement: Supplementary file 4 — Additional file 4. Preplanned tables and figures. [file 13104_2018_3481_MOESM4_ESM.docx]

**PREPLANNED TABELS AND FIGURES**

**Figure 1: CONSORT diagram:**

**Table 1: Baseline characteristics**

|  | **Spot sign positive** | | **Spot sign negative** | |
| --- | --- | --- | --- | --- |
|  | **Tranexamic aid** | **Placebo** | **Tranexamic acid** | **Placebo** |
| Patients randomised |  |  |  |  |
| Age (years)*, Mean (SD) |  |  |  |  |
| >70 years, (%) |  |  |  |  |
| Sex*, Male (%) |  |  |  |  |
| Ethnic origin, (%) |  |  |  |  |
| White |  |  |  |  |
| Black |  |  |  |  |
| South Asian |  |  |  |  |
| East Asian |  |  |  |  |
| Other Asian |  |  |  |  |
| Other |  |  |  |  |
| Time from onset to baseline CCT, Median [IQR] |  |  |  |  |
| Time from onset to randomisation (hours)*, Median [IQR] {range} |  |  |  |  |
| >3 hours (%) |  |  |  |  |
| >4.5 hours (%) |  |  |  |  |
| Time from onset to first treatment-dosage (hours), median [IQR] {range} |  |  |  |  |
| All randomised treatment received as allocated |  |  |  |  |
| History of antiplatelet therapy on admission*, (%) |  |  |  |  |
| History of statin use prior to admission, (%) |  |  |  |  |
| History of previous ischemic stroke or TIA, (%) |  |  |  |  |
| History of ischaemic heart disease, (%) |  |  |  |  |
| History of thromboembolism, (%) |  |  |  |  |
| Pre-stroke modified Rankin score, median [IQR] {range} |  |  |  |  |
| >2, (%) |  |  |  |  |
| GCS, mean (SD) {range} |  |  |  |  |
| <8, (%) |  |  |  |  |
| NIHSS score*, mean (SD) {range} |  |  |  |  |
| >13, (%) |  |  |  |  |
| SBP (mmHg)*, mean (SD) {range} |  |  |  |  |
| >170 mmHg, (%) |  |  |  |  |
| DBP (mmHg), mean (SD) {range} |  |  |  |  |
| Haematoma location, (%) |  |  |  |  |
| Supra-tentorial Lobar |  |  |  |  |
| Supra-tentorial Deep |  |  |  |  |
| Infra-tentorial |  |  |  |  |
| Combination of above locations |  |  |  |  |
| Intraparenchymal haematoma volume, (%) |  |  |  |  |
| < 30 mL |  |  |  |  |
| 30 to 60 mL |  |  |  |  |
| - 60mL |  |  |  |  |
| Intraparenchymal haematoma volume (ml), mean (SD) {range} |  |  |  |  |
| Intra-ventricular haemorrhage*, (%) |  |  |  |  |
| Intraventricular haematoma volume (ml), mean (SD) {range} |  |  |  |  |
| Subarachnoid haemorrhagic extension, (%) |  |  |  |  |

*Minimisation criteria

TXA: Tranexamic acid; SD: Standard deviation; IQR: interquartile range; TIA: transitory ischaemic attack; IDH: Ischaemic heart disease; mRS: modified Rankin Scale; GCS: Glasgow Coma Scale; NIHSS: National Institute of Health Stroke Scale; SBP: Systolic blood pressure; DBP; Diastolic blood pressure; CTA: Computed Tomography Angiography.

Table 2: Primary outcome.

| Variable | | Tranexamic acid | Placebo | OR/MD | aOR/aMD | P for homogeneity |
| --- | --- | --- | --- | --- | --- | --- |
| **Primary outcome** | | | | | |  |
| 24-hour CCT intraparenchymal haematoma volume – median (IQR) | | | | | |  |
| Spot sign positive | |  |  |  |  |  |
| Spot sign negative | |  |  |  |  |  |
| Combined 24-hour intraparenchymal and intraventricular haematoma volume – median (IQR) | | | | | |  |
| Spot sign positive | |  |  |  |  |  |
| Spot sign negative | |  |  |  |  |  |
| **Secondary outcome** | | | | | |  |
| Composite secondary outcome – no./total no. (%) | | | | | |  |
| Spot sign positive | |  |  |  |  |  |
| Spot sign negative | |  |  |  |  |  |
| Individual components of the composite secondary outcome | | | | | | |
|  | Significant intraparenchymal haematoma expansion – no./total no. (%) | | | | |  |
|  | Spot sign positive |  |  |  |  |  |
|  | Spot sign negative |  |  |  |  |  |
|  | Delayed intraventricular or subaracnoid haemorrhagic extension – no./total no. (%) | | | | |  |
|  | Spot sign positive |  |  |  |  |  |
|  | Spot sign negative |  |  |  |  |  |
|  | Significant intraventricular haematoma expansion – no./total no. (%) | | | | |  |
|  | Spot sign positive |  |  |  |  |  |
|  | Spot sign negative |  |  |  |  |  |
|  | Early neurological deterioration within 24 hours – no./total no. (%) | | | | |  |
|  | Spot sign positive |  |  |  |  |  |
|  | Spot sign negative |  |  |  |  |  |

OR: Odds ratio; MD: Mean difference; BLR: bivariate logistic regression; MLR: multivariate linear regression.

Table 3: Secondary outcomes.

| Variable | Tranexamid acid | Placebo | OR/MD | aOR/aMD | P for homogeneity |
| --- | --- | --- | --- | --- | --- |
| Any serious adverse event (at least one reported within 28 days) -- no./total no. (%) | | | | |  |
| Spot sign positive |  |  |  |  |  |
| Spot sign negative |  |  |  |  |  |
| Any safety outcome (at least one reported within 28 days) -- no./total no. (%) | | | | |  |
| Spot sign positive |  |  |  |  |  |
| Spot sign negative |  |  |  |  |  |
| Any thromboembolic event (at least one reported within 28 days) – no./total no. (%) | | | | |  |
| Spot sign positive |  |  |  |  |  |
| Spot sign negative |  |  |  |  |  |

Table 4: Day-90 outcomes.

| Variable | No. available for analysis | TXA | Placebo | OR/MD/HR | | aOR/aMD/aHR | P for homogeneity |
| --- | --- | --- | --- | --- | --- | --- | --- |
| Modified Rankin Scale at day-90 (dichotomous mRS 4-6), -- no./total no. (%) | | | | | | |  |
| Spot sign positive |  |  |  |  |  | |  |
| Spot sign negative |  |  |  |  |  | |  |
| Barthel index at day-90, median (IQR) | | | | | | |  |
| Spot sign positive |  |  |  |  |  | |  |
| Spot sign negative |  |  |  |  |  | |  |
| Death by day-90, median (IQR) | | | | | | |  |
| Spot sign positive |  |  |  |  |  | |  |
| Spot sign negative |  |  |  |  |  | |  |

OLR: ordinal logistic regression; MLR: multivariate linear regression; CPHR: Cox Proportional Hazard model.
